# Preparation and Characterization of Inclusion Complexes of *β*-Cyclodextrin and Phenolics from Wheat Bran by Combination of Experimental and Computational Techniques

**DOI:** 10.3390/molecules25184275

**Published:** 2020-09-18

**Authors:** Tuba Simsek, Bakhtiyor Rasulev, Christian Mayer, Senay Simsek

**Affiliations:** 1 Department of Physical Chemistry, Duisburg-Essen University, Universitätsstr. 2, 45141 Essen, Germany; tuba.simsek1202@gmail.com; 2 Department of Plant Sciences, North Dakota State University, Fargo, ND 58102, USA; 3 Department of Coatings and Polymeric Materials, North Dakota State University, Fargo, ND 58102, USA; bakhtiyor.rasulev@ndsu.edu

**Keywords:** cyclodextrin, wheat, phenolics, bioactive compounds, inclusion complex, interaction

## Abstract

Bitterness often associated with whole wheat products may be related to phenolics in the bran. Cyclodextrins (CDs) are known to form inclusion complexes. The objective was to form inclusion complexes between *β*-CD and wheat phenolics. Pure phenolic acids (trans-ferulic acid (FA), caffeic acid (CA), and *p*-coumaric acid (CO)) and phenolic acids from wheat bran were used to investigate complex formation potential. Complexes were characterized by spectroscopy techniques, and a computational and molecular modeling study was carried out. The relative amount of complex formation between *β*-CD and wheat bran extract was CA > CO > FA. The phenolic compounds formed inclusion complexes with *β*-CDs by non-covalent bonds. The quantum-mechanical calculations supported the experimental results. The most stable complex was CO/*β*-CD complex. The Δ*H* value for CO/*β*-CD complex was −11.72 kcal/mol and was about 3 kcal/mol more stable than the other complexes. The QSPR model showed good correlation between binding energy and ^1^H NMR shift for the H^5^ signal. This research shows that phenolics and *β*-CD inclusion complexes could be utilized to improve the perception of whole meal food products since inclusion complexes have the potential to mask the bitter flavor and enhance the stability of the phenolics in wheat bran.

## 1. Introduction

Phenolic compounds are a part of the human diet found in many plant foods, such as wheat and other cereal grains. Phenolic acids are a category of phenolic compounds that are classified as benzoic or cinnamic acids. Trans-ferulic acid (FA), *p*-coumaric acid (CO), and caffeic acid (CA) are cinnamic acids, and they are most commonly found in cereal grains. The cinnamic acids are concentrated in the outer layers of the grain [1,2]. Cinnamic acids have at least one aromatic ring with one or more hydroxyl groups in different positions and with one carboxyl group. Phenolic acids are effective antioxidant, antimutagenic, and anticancerogenic compounds. They can react with free radicals and reactive oxygen species, and the ability of phenolic acids to scavenge reactive oxygen species (ROS) is beneficial for human health [3,4]. However, hydroxycinnamic acids are responsible for bitter flavors [5,6] and can influence the bitter taste in whole wheat products.

Cyclodextrins (CDs) are known for their ability to form inclusion complexes based on their conical shape [7,8]. CDs have a hydrophobic inner cavity with the inner protons H^3^ and H^5^ and a hydrophilic outer surface with the outer protons H^1^, H^2^, H^4^, and H^6^. The secondary hydroxyl groups are situated on the wide rim, and the primary hydroxyl groups are placed on the narrow rim [9,10,11,12]. CDs are classified as *α*-, *β*-, and *γ*-CD with six, seven, and eight glucose units, respectively. The CDs have different cavity diameters based on the number of glucose units in their structure [13]. The cavity diameter is a parameter for inclusion complex formation because the organic molecule (called guest) must fit into the CDs cavity based on their geometry and size comparable to the lock and key principle. Additionally, the guest must be hydrophobic due to the hydrophobic cavity of the CD. The more hydrophobic the guest is, the stronger the interaction between guest and host [14].

An essential criterion for food choice is good taste since food products have a fundamental requirement to remain in competitive markets. Sensory characteristics of wholegrain products are described as grainy, bitter, or astringent [15]. Bitter tastes in wholegrain, bran, and germ are unfavorable. Studies have shown that sensory panelists found whole wheat bread to be more bitter than white bread [16,17]. Another sensory evaluation study showed differences in bitterness for whole wheat products made from red wheat vs. white wheat [18]. Taste thresholds for phenolic acids have been determined to be in the range of 5–90 ppm, depending on the specific phenolic acid. The presence of multiple phenolic acids has a synergistic effect on the taste threshold [18]. Free, conjugated, and bound phenolics have been determined in the ranges of 3–30 ppm, 76–337 ppm, and 208–964 ppm, respectively [19], which are within the threshold of detection by taste. Sensory analysis has also shown that phenolic content impacts the flavor of bread and crackers made from red or white whole wheat flours [16]. A solution for masking bitterness could be the inclusion complex formation of phenolic acids with CDs [20].

Controlled release and stabilization of phenolic acids is another benefit to complexation with *β*-CD. Phenolic acids, such as ferulic acid, are important antioxidants found in wheat bran [3]. Phenolic acids have low water solubility, but complexation with *β*-CD would increase their solubility. The antioxidant capacity of ortho and meta-coumaric acid has been seen to increase when complexed with *β*-CD [21]. Furthermore, a complexation with *β*-CD led to a three-fold increase in the solubility of ferulic acid [22]. Ferulic acid, the main phenolic acid in wheat, has been shown to have the greatest antioxidant capacity due to its structural features contributing to high free radical scavenging potential [23]. Ferulic acid has also been shown to be efficiently absorbed in the intestine [23], so stabilization of ferulic acids in wheat bran and whole wheat products could be highly beneficial. It is important for phenolic acids to reach the colon intact in order for them to be functional as antioxidants and anti-inflammatory compounds [23].

The objective of this study was to form inclusion complexes between CDs and phenolics from wheat bran. This study intended to investigate how the inclusion complexes will form when phenolic acids are included in a mixture and an initial study of inclusion complex formation with phenolic acids extracted from wheat bran. The complex mixtures were prepared between CO, CA, FA, and *β*-CD because these three phenolic acids are commonly found in wheat bran. Although other phenolics are found in wheat bran, these three were chosen as a representative selection. Free phenolic compounds were also extracted from wheat bran and the complexation formed with the wheat bran extract and *β*-CD was studied. The inclusion complex formation is advantageous for possible applications in the food industry due to the alteration of the physicochemical properties of the phenolic acids. The phenolic acids CDs complexes have higher solubility, durability, and stability. Additionally, bitter tastes and unpleasant odors could be eliminated. Furthermore, the guest molecules will release under controlled conditions [14,24]. These changes to the properties of the included guest would allow for additional applications in whole wheat products.

## 2. Results and Discussion

In the present study, the aim was application of cyclodextrins for removal of bitter phenolic acid compounds in wheat bran. Various complex mixtures were studied as a simulation for the mixture of phenolics in wheat bran. The complex mixtures between CO, CA, FA, and *β*-CD, were prepared because these three phenolic acids are commonly found in wheat bran. The mixtures were prepared as follows 1. Mixture: CO, CA, *β*-CD, 2. Mixture: CO, FA, *β*-CD, 3. Mixture: CA, CO, *β*-CD, 4. Mixture: CA, FA, CO, *β*-CD. The physicochemical parameters were characterized for possible complex formation in the mixtures by nuclear magnetic resonance spectroscopy (NMR), mass spectroscopy (MS), Fourier-transform infrared spectroscopy (FT-IR) and differential scanning calorimetry (DSC).

Further investigation was carried out utilizing a phenolic extract mixture from wheat bran. A sample was extracted from wheat bran by the method of Kim, et al. [25]. The extract contained seven phenolic acids as follows: CA, FA, CO, vanillic acid, sinapic acid, hydroxybenzoic acid, and syringic acid. The extract was mixed with *β*-CD and characterized by high-performance liquid chromatography (HPLC), NMR, MS, FT-IR, and DSC.

### 2.1. HPLC Studies—Wheat Bran Extract

The wheat bran extract was analyzed by HPLC to determine the composition of phenolic acids. The wheat bran extract sample was analyzed and compared with two sets of phenolic acid standards at 280 nm and 320 nm. The wheat bran extract sample contained seven phenolic acids as follows: CA (0.31 μg/mL), FA (7.86 μg/mL), CO (0.46 μg/mL), vanillic acid (8.08 μg/mL), sinapic acid (1.50 μg/mL), hydroxybenzoic acid (1.67 μg/mL), and syringic acid (6.68 μg/mL). Overall, the wheat bran extract contained the most common phenolic acids found in wheat bran, CA, FA, and CO, for which the present work is focused on.

### 2.2. H Nuclear Magnetic Resonance Studies

#### 2.2.1. NMR—Synthetic Mixtures

**Figure 1:**
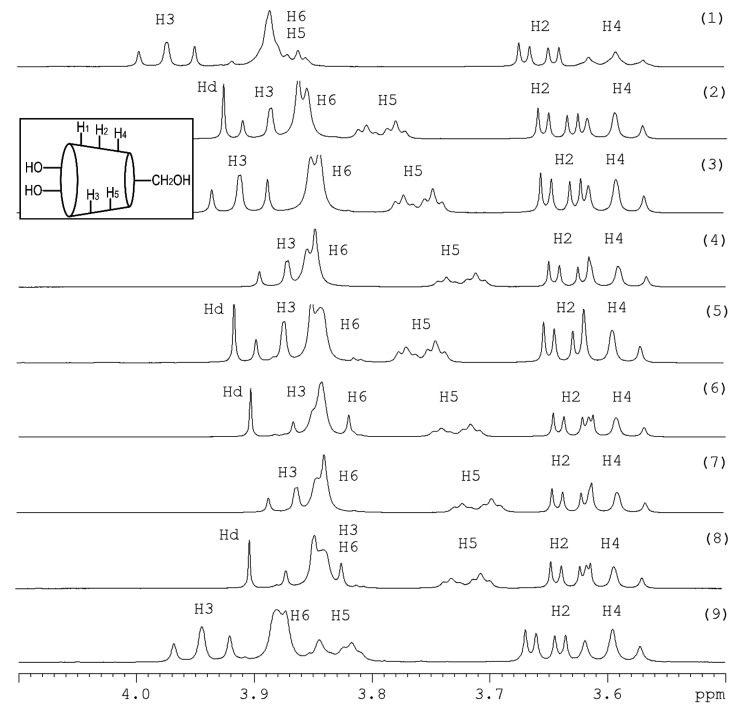
^1^H-NMR-Spectra (400 MHz): (1) *β*-cyclodextrin, (2) complex of *β*-cyclodextrin and trans-ferulic acid, (3) complex of *β*-cyclodextrin and caffeic acid, (4) complex of *β*-cyclodextrin and *p*-coumaric acid, (5) complex of *β*-cyclodextrin, caffeic acid, and trans-ferulic acid, (6) complex of *β*-cyclodextrin, *p*-coumaric acid, and trans-ferulic acid, (7) complex of *β*-cyclodextrin, caffeic acid, and *p*-coumaric acid, (8) complex of *β*-cyclodextrin, caffeic acid, trans-ferulic acid, *p*-coumaric acid, (9) wheat bran extract with *β*-cyclodextrin each 5 mM in deuterium oxide in the range of 4.10 ppm to 3.50 ppm. Hd is the shift from the hydrogen in the deuterium oxide.

Firstly, synthetic mixtures were studied by NMR. The chemical shifts for the inclusion complexes and free components were determined, and the chemical shift differences were calculated by the Equation (1):

∆δ = δ_pure_ − δ_complex_ (1)

where δ_pure_ is the chemical shift for the free components, and δ_complex_ is the chemical shift for the inclusion complex. The chemical shift differences show the interaction between the phenolic acids and *β*-CD and the position of the phenolic acid inside or outside the cavity of *β*-CD. The ^1^H-NMR spectra are shown in Figure 1. The results demonstrated that the phenolic acids -CA, FA, and CO in the synthetic mixtures were included by *β*-CD based on the chemical shift differences of both inner protons H^5^ and H^3^. The peak H^3^ had been shifted slightly. The peak H^5^ had been completely separated from the peak H^6^ and shifted to different degrees for all complexes. Additionally, the chemical shift value for the proton H^5^ is specific to each phenolic acid, which is included in the cavity of *β*-CD. In the synthetic mixture -CA, FA, *β*-CD, the chemical shift value for the proton H^5^ is comparable to the CA/*β*-CD-complex since the peak H^5^ had been shifted from 3.8579 ppm to 3.7578 ppm. In the synthetic mixture -CO, FA, *β*-CD, the chemical shift value for the peak H^5^ is attributed to the CO/*β*-CD-complex because the peak H^5^ was shifted from 3.8579 ppm to 3.7290 ppm. In the synthetic mixture -CA, CO, *β*-CD, the chemical shift differences of the proton H^5^ are comparable with the CO/*β*-CD-complex. The peak H^5^ at 3.857 ppm had been shifted to 3.709 ppm. In the last synthetic mixture -CA, FA, CO, *β*-CD, the chemical shift differences of the peak H^5^ is attributed to the *p*-coumaric/*β*-cyclodextrin-complex since the peak H^5^ at 3.857 ppm had been shifted to 3.717 ppm. The complexation of the phenolic acids -CA, FA, and CO was confirmed with *β*-CD. Additionally, we can see that the phenolic acids CO had the highest potential to include in the cavity of *β*-CD in all synthetic mixtures.

#### 2.2.2. NMR Studies—Wheat Bran Extract

The wheat bran extract complex was characterized by NMR (Figure 1). The spectrum contained the characteristic peaks for *β*-CD. The peaks were attributed as follows: 3.876 ppm for H^6^, 3.563 ppm for H^5^, 3.596 ppm for H^4^, 3.563 ppm for H^3^, 3.652 ppm for H^2^, and 5.074 ppm for H^1^. The peak H^5^ had been completely separated and the peak H^6^ was visible as a separate peak and had been shifted slightly from 3.5630 ppm to 3.832 ppm. The peak H^3^ had been shifted radically from 3.5630 ppm to 3.945 ppm. All changes demonstrated that the phenolic acids in wheat bran extract were included in the cavity of *β*-CD. Both inner protons H^3^ and H^5^ were shifted and showed the formation of the inclusion complexes. The effective complexation of the wheat bran free phenolic acids is important for mitigation of their bitter flavors. Additionally, the complexation can stabilize the free phenolic acids and improve their antioxidant capacity [21,22].

### 2.3. Mass Spectroscopy Studies

#### 2.3.1. MS—Synthetic Mixtures

**Figure 2:**
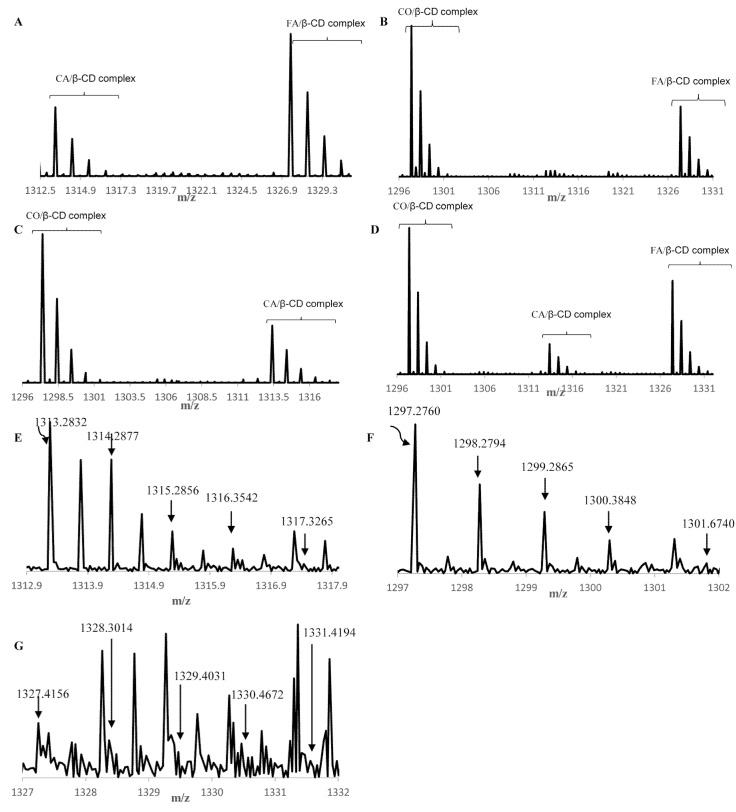
MS spectra: (**A**) caffeic acid, trans-ferulic acid, and *β*-cyclodextrin mixture, (**B**) *p*-coumaric acid, trans-ferulic acid and *β*-cyclodextrin mixture, (**C**) *p*-coumaric acid, caffeic acid, and *β*-cyclodextrin mixture, (**D**) *p*-coumaric acid, caffeic acid, trans-ferulic acid and *β*-cyclodextrin mixture, (**E**) extract in the range 1312.0 and 1317.0 m/z—caffeic acid/*β*-cyclodextrin-complex, (**F**) extract in the range 1297 and 1302 m/z—*p*-coumaric acid/*β*-cyclodextrin-complex, and (**G**) extract in the range 1327 and 1332 m/z—trans-ferulic acid/*β*-cyclodextrin-complex.

Secondly, synthetic mixtures were investigated by MS. In all synthetic mixtures, we can see that the complexes were formed with all phenolic acids in different amounts, based on total peak height and signal intensity (Figure 2).

In the synthetic mixture -CA, FA, *β*-CD, both complexes were formed in different amounts. The amount of the FA/*β*-CD-complex was higher than the CA/*β*-CD-complex. In the synthetic mixture -CO, FA, *β*-CD, the CO/*β*-CD-complex was formed in a higher amount than the FA/*β*-CD-complex. In the synthetic mixture -CO, CA, *β*-CD, the amount for the CO/*β*-CD-complex was higher than the CA/*β*-CD-complex. In the synthetic mixture -CO, CA, FA, *β*-CD, the CO/*β*-CD-complex was more than FA/*β*-CD- and CA/*β*-CD-complexes. Overall, all phenolic acids were included in the cavity of *β*-CD in different amounts. The phenolic acid CO was included in a higher amount than CA and FA. The MS data shows that when forming complexes between *β*-CD and a mixture of phenolic acids the amount of complex formation will not be equal for each phenolic acid in the mixture. However, the complexes between *β*-CD and each phenolic acid formed in different amounts depending on which phenolic acids were present. For the synthetic mixture of phenolic acids, the relative amount of complex formation between *β*-CD and caffeic acid, ferulic acid, and coumaric acid was CO > FA > CA.

#### 2.3.2. MS—Wheat Bran Extract

The wheat bran complex was investigated by MS. The spectrum contained the characteristic peaks for the three complexes as follows: FA/*β*-CD-complex, CA/*β*-CD-complex, and CO/*β*-CD-complex. The peaks at 1313.2832, 1314.2877, 1315.2856, 1316.3542, and 1317.3265 m/z were attributed to the CA/*β*-CD-complex. The peaks at 1297.2760, 1298.2794, 1299.2865, 1300.384,8 and 1301.6740 m/z are assigned to the CO/*β*-CD-complex. The peaks at 1327.4156, 1328.3014, 1329.4031, 1330.4672 and 1331.4194 m/z were attributed to the FA/*β*-CD-complex. All three complexes were present in the wheat bran extract mixture with *β*-CD. All MS spectra are shown in Figure 2. The wheat bran extract mixture with *β*-CD contained all three complexes in different amounts. Differing to the results seen for the synthetic mixture, the relative amount of complex formation between *β*-CD and caffeic acid, ferulic acid and coumaric acid in the wheat bran extract was CA > CO > FA. This may be due to the presence of other phenolic acids, such as vanillic acid or other compounds in the wheat bran extract.

### 2.4. Fourier-Transform Infrared Spectroscopy Studies

#### 2.4.1. FT-IR—Synthetic Mixtures

**Figure 3:**
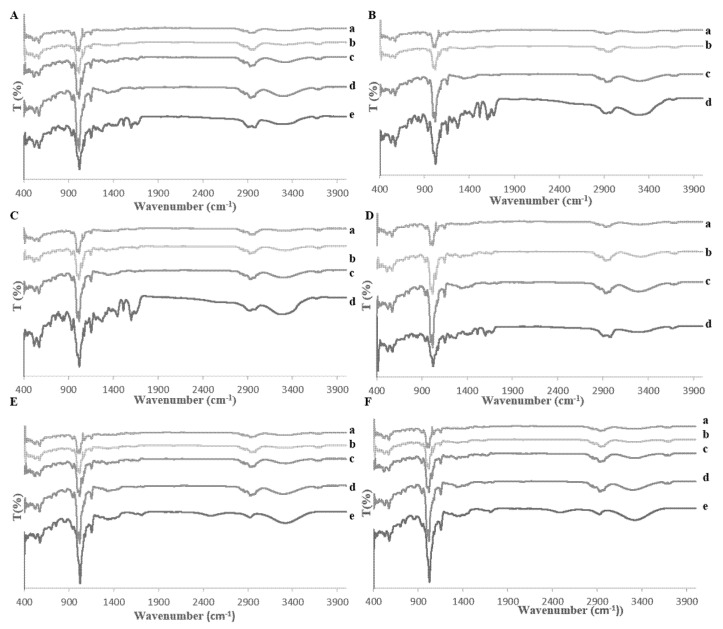
Fourier-transform infrared spectroscopy graphs: (**A**) (a) trans-ferulic acid, (b) caffeic acid, (c) *p*-coumaric acid, (d) *β*-cyclodextrin, (e) mixture, (**B**) (a) trans-ferulic acid, (b) caffeic acid, (c) *β*-cyclodextrin, (d) mixture, (**C**) (a) caffeic acid, (b) *p*-coumaric acid, (c) *β*-cyclodextrin, (d) mixture, (**D**) (a) trans-ferulic acid, (b) *p*-coumaric acid, (c) *β*-cyclodextrin, (d) mixture; (**E**) (a) trans-ferulic acid, (b) caffeic acid, (c) *p*-coumaric acid, (d) *β*-cyclodextrin, (e) mixture; (**F**) (a) trans-ferulic acid, (b) caffeic acid, (c) *p*-coumaric acid, (d) *β*-cyclodextrin, and (e) extract and *β*-cyclodextrin mixture.

The behavior of the synthetic mixtures was characterized by FT-IR. The spectrum of *β*-CD had the characteristic peaks for the hydroxyl groups at 3345 cm^−1^, for the C-H stretching vibrations at 2950 cm^−1^, and for the C-O stretching vibration at 1019, 938, 571, and 514 cm^−1^. In the spectra of pure phenolic acids -CA, CO, and FA, the peak at around 3345 cm^−1^ appeared for the hydroxyl groups and the peak at around 2937 cm^−1^ was for the C-H stretching vibrations. The peak at around 1649 cm^−1^ occurred for the aromatic conjugated carbonyl. The peaks at around 1459, 1412, 1370, 1366, 1324, and 1291 cm^−1^ are attributed to the aromatic ring of the phenolic acids. The peak at around 1019 cm^−1^ illustrated the C-O stretching vibration. The peaks at around 526, 596, 702, and 757 cm^−1^ corresponded to the four hydrogen atoms on the phenyl ring of C-O. The spectra of all mixtures showed differences in intensity since the peak at around 1019 cm^−1^ was decreased; the peak at around 3345 cm^−1^ increased in intensity and had been shifted to around 3330 cm^−1^. All FT-IR spectra are shown in Figure 3. Overall, all changes demonstrated that the phenolic acids in the mixtures were included within the cavity of *β*-CD. Successful complex formation is beneficial for increasing the free radial scavenging capacity of phenolic acids, such as caffeic acid and ferulic acid [22].

#### 2.4.2. FT-IR—Wheat Bran Extract

The wheat bran complex was studied by FT-IR. In the FT-IR spectrum of the pure phenolic acids, the peak at 3345 cm^−1^ attributed to the hydroxyl groups, and the peaks at around 1649 cm^−1^ occurred for the aromatic conjugated carbonyl. The peak at 2937 cm^−1^ was attributed to the C-H stretching vibrations, and the peaks at around 1459, 1412, 1370, 1366, 1324, and 1291 cm^−1^ were characteristic of the aromatic ring of the phenolic acids. The peaks at around 1019 cm^−1^ were attributed to the C-O stretching vibration and the peaks at around 526, 596, 702, and 757 cm^−1^ were associated with the four hydrogen atoms on the phenyl ring of C-O. In the spectrum of *β*-CD, the peak at 3345 cm^−1^ appeared for the hydroxyl groups, the peak at 2950 cm^−1^ occurred for the C-H stretching vibration and the peaks at 1019, 938, 571, and 514 cm^−1^ were attributed to the C-O stretching vibration. In the spectrum of the complex, the peak at 1019 cm^−1^ had been decreased; the peak at 3345 cm^−1^ had increased in intensity and shifted to 3330 cm^−1^. All FT-IR spectra are shown in Figure 3. The complexation affects the IR activity of the phenolic acids. The bands of the included phenolic acid are shifted, or their intensity influenced, while the spectrum of the *β*-CD remains mostly unchanged.

### 2.5. Differential Scanning Calorimetry Studies

#### 2.5.1. DSC—Synthetic Mixtures

Finally, the thermal behavior of the synthetic mixtures was studied by DSC (data not shown). The free phenolic acids -CA, FA, CO have a characteristic peak corresponding to their melting point. The free phenolic acid -FA has a peak at 173 °C. The phenolic acids -CO and CA have a peak at 221 °C. The thermal curve of *β*-CD showed a broad peak at 67 °C and a small peak at 223 °C. The thermal curves of the synthetic mixtures demonstrated wide variation in thermal behavior as compared to pure compounds since the peak for the melting point was not present in complexes, and the peak of *β*-CD had been shifted and increased in intensity. All changes showed that the phenolic acids in the mixture were included in the cavity of *β*-CD. The change of the melting point may be attributed to the complex formation between the *β*-CD and the free compounds. In the complex, the included phenolic acid and the host *β*-CD had the same melting point.

#### 2.5.2. DSC Studies—Wheat Bran Extract

The wheat bran complex was investigated by DSC (data not shown). All three pure phenolic acids showed a characteristic peak corresponding to their melting point as followed the peak at 173 °C for FA, at 221 °C for CO, and at 221 °C for CA. In the thermal curve of *β*-CD, a broad peak was visible at 67 °C, and a small peak was visible at 223 °C. In the thermal curve of the complex, we can see that the thermal curve did not have similar thermal behavior to pure compounds. The melting point peaks seen for the pure compounds were no longer present. The peak of pure *β*-CD had been shifted and increased in intensity. All changes between the pure compounds and the complex demonstrated that all phenolic acids in the extract mixture were included in the cavity of *β*-CD. The change in melting point indicated successful complex formation. The included phenolic acid had the same melting point as the host *β*-CD.

### 2.6. Computational Analysis

We performed a combined computational study involving molecular modeling and quantitative structure–property relationship modeling (QSPR) [26,27]. To confirm the experimental results and understand the influence of the structural properties of phenolic acids on interaction with *β*-CD, we computed each structure of phenolic acids quantum-chemically to get important energy related properties. Here, applying the PM6 method [28] we looked at the lowest energy structures of inclusion systems. For each complex, we calculated the binding energy or enthalpy changes (Δ*H*). The following equation (Equation (2)) was used to calculate the energies for each complex:

Δ*H_enthalpy_* = Δ*H_FLAVOR/CD inclusion complex_* − (Δ*H_CD_* + Δ*H_flavor_*) (2)

Furthermore, the Δ*H_f_* values for mixture systems were calculated based on Δ*H_f_* values of pure phenolic acids. For this, the equation that takes a mean value of two or three compounds was applied. For example, for two phenolic acids’ system the following equation is applied: Δ*H_f_*_(mix)_ = (Δ*H_f__flavor1_* + Δ*H_f flavor2_*)/2. The values of each computed property for each inclusion complex, as well as calculated Δ*H_f_* values for mixture systems, are reported in Table 1A.

It was discussed in our previous study that according to the equation, the more negative the enthalpy change is, the stronger the interaction between host and guest molecules [29]. From the obtained data, it was observed that the most stable complex is CO/*β*-CD complex among all three complexes, including CA/*β*-CD and FA/*β*-CD. Thus, Δ*H* value for CO/*β*-CD complex is −11.72 kcal/mol, which is about 3 kcal/mol more stable than CA/*β*-CD and FA/*β*-CD complexes. This is mostly consistent with the relative amounts of complex formation seen for the MS spectra of the synthetic mixture of the phenolic acids. The relative amount of complex formation determined by MS was CO > FA > CA. However, the computational study predicted that the relative complex stability order would be CO > CA > FA. There is only a small difference in the Δ*H* values of CA and FA, so there may be some other factors affecting the actual complex formation. Additionally, it was previously discussed that negative values for the Δ*H_f_* indicate that the formation of all the complexes are exothermic processes and all complexes are relatively stable. It can be stated from the computational data that the hydrogen bond between the hydrogen atoms of *β*-CD and oxygen atoms of phenolic acids strengthen the host-guest association in these complexes. It was also confirmed before that the smaller size of the CO molecule makes it a better fit within the cavity of *β*-CD than bulky CA and FA molecules, which improves the stability of CO/*β*-CD complex.

**Figure 4:**
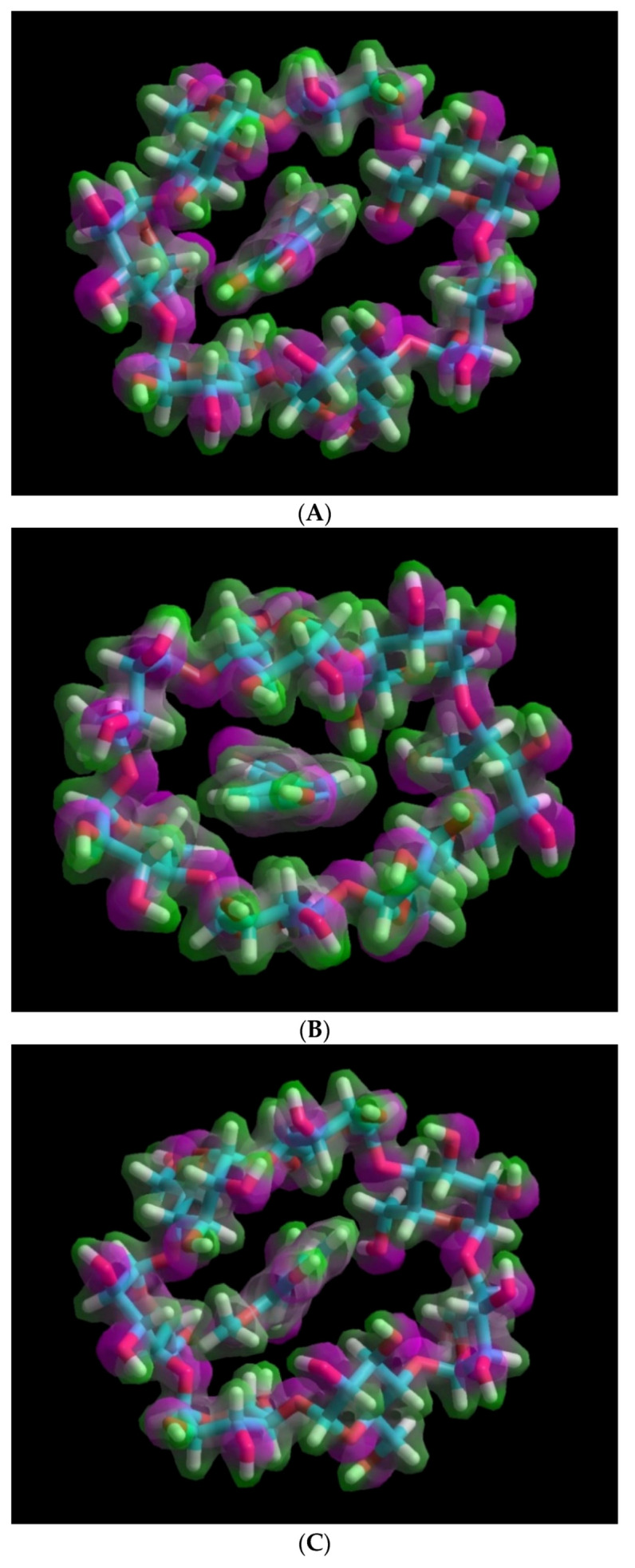
Electrostatic surface potential (ESP) representation and phenolic acid positions within the CD: (**A**) caffeic acid and *β*-cyclodextrin, (**B**) *p*-coumaric acid and *β*-cyclodextrin, (**C**) trans-ferulic acid and *β*-cyclodextrin.

The molecular orbital energy gaps (HLgap) that were calculated based on the higher occupied molecular orbital (HOMO) and lower unoccupied molecular orbital (LUMO) energies of the inclusion complexes also confirmed the stability trend. Thus the HLgap of the CO/*β*-CD inclusion complex has the largest value (8.19 eV) compared to that of other complexes (CA/*β*-CD and FA/*β*-CD), Table 1B. In addition, the electrostatic surface potentials (ESP) for the investigated complexes are shown in Figure 4. The ESP surface represents the charges distribution over the surface of interacting molecules, for CA/*β*-CD complex (Figure 4A), CO/*β*-CD complex (Figure 4B), and FA/*β*-CD complex (Figure 4C).

Next, we investigated the influence of Δ*H_f_* values of all complexes, including the complexes with mixture systems, where two or more phenolic acids were involved. According to the 1H NMR spectra, there were significant changes in the H5 signal shifts according to the nature of the complex. We found that the 1H NMR shifts were in good correlation with the binding energy, i.e., with computationally obtained Δ*H_f_* for the complex systems, including mixture systems. The Δ*H_f_* value for the mixture systems was calculated using PM6 quantum-chemical method and converted according to the modified Equation (2), as was discussed before. Then, based on calculated Δ*H_f_* values for phenolic acids and their mixtures, the predictive QSPR model was developed that builds a relationship between calculated binding energy of the complex and the experiment H5 chemical shift (a measure of the complexation strength between CD and a phenolic compound). The following linear regression model was obtained:

δ(H5) = 0.029 (± 0.022) Δ*H_f_* + 4.042 (± 0.225) (3)

*N* = 7, *r* = 0.837, *r^2^* = 0.701, *s* = 0.017, *p* = 0.018, *F* = 11.735

Where δ(H5) is the 1H NMR shift in ppm for H5, Δ*H_f_*-binding energy for the complex, *N*-number of data points, *r* and *r^2^* are correlation coefficients between observed and predicted values (where *r^2^* is a squared correlation coefficient), *s*-standard error of estimation, *F* is the *F*-ratio between the variances of observed and calculated property, and *p*-probability value for calculated *F*.

**Figure 5:**
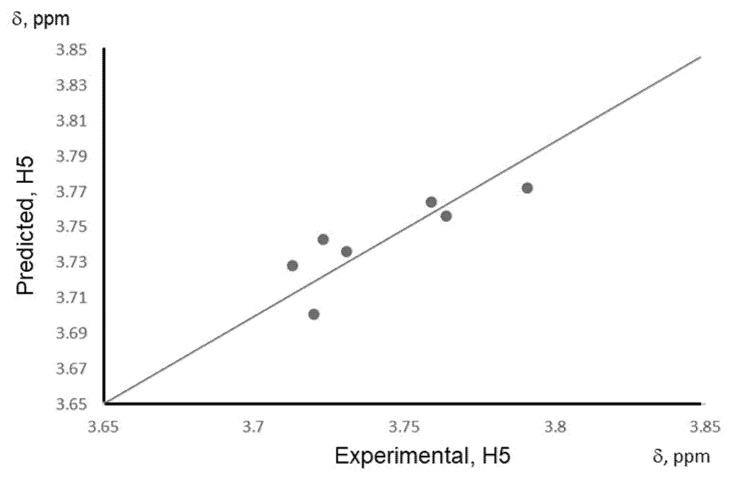
A correlation plot of experimental and predicted values of δ (H^5^) based on the QSPR model for pure phenolic acids and their mixtures with *β*-cyclodextrin.

The QSPR model shows a good correlation between the binding energy and the ^1^H NMR shift for the H^5^ signal (Equation (3) and Figure 5). The model is tuned mainly for currently investigated phenolic acid/*β*-CD systems, but with an increased pool of experimental data, this model can be extended to predict H^5^ chemical shift for many other chemical compounds to bind with *β*-CD and then for an inverse prediction: to predict what mixture of certain phenolic acids is presented in the solution, based on NMR spectrum of this mixture with *β*-CD. The developed QSPR model is able to predict the strength of the complexation for most of the investigated molecules with at least 70% accuracy (*r*^2^ = 0.701). Overall, the combination of computational studies was able to provide understanding in experimental findings, for example in trends in chemical shifts for the NMR spectra of CD‘s protons, responsible for interacting with the phenolic acid molecule, as well as finding the relationship with binding energy and H^5^ chemical shifts of pure phenolic acids and mixture systems. The developed QSPR model can be beneficial in assessing the type of phenolic acid mixture in the solution, as well as the concentration of presented phenolic acid compounds in the solution, with the help of additional concentration experiments.

## 3. Experimental

### 3.1. Materials

Caffeic acid (3,4-dihydroxy-cinnamic acid, CA), trans-ferulic acid (4-hydroxy-3-methoxy-cinnamic acid, FA), *p*-coumaric acid (4-hydroxy-cinnamic acid, CO), and *β*-cyclodextrin (*β*-CD) were purchased from Sigma Aldrich in analytical grade. Wheat bran was obtained from the North Dakota State University Wheat Quality Laboratory (Department of Plant Science). Wheat bran was produced by milling a sample of the Glenn variety hard red spring wheat on a Buhler ML-202 laboratory mill. The bran fraction was then used for phenolic extraction.

### 3.2. Extraction of Free Phenolic Acids

Free phenolic acids were extracted according to the method of Kim, Tsao, Yang and Cui [25]. Wheat bran was milled and sieved. The fine bran (200 g) was put into an Erlenmeyer flask and defatted with hexane at a 4:1 ratio. The sample was mixed with a mechanical shaker for 1 h at room temperature. The mixture was filtered by Whatman No. 1 filter paper. The final bran was dried in a hood at room temperature. The filtrate was evaporated and the residue was weighed. The defatted bran residue was stirred with 80% methanol at a 5:1 ratio (v/w) for 1 h at room temperature. The sample was filtered with Whatman No. 1 filter paper. The methanol fraction was reserved, and the solid fraction of bran residue was stirred a second time with 80% methanol at a 5:1 ratio (v/w) for 1 h at room temperature. The methanol fractions were combined, and the solvent was evaporated at 40 °C by a rotary evaporator. The remaining extract was freeze-dried and stored in a sealed container at 0 °C prior to use.

### 3.3. Analysis of Free Phenolic Acid Extract

The phenolic acids were analyzed using high performance liquid chromatography (HPLC) according to the method of *Kim, Tsao, Yang and Cui* [25], with some modifications. An Agilent 1200 HPLC system with variable wavelength detector (VWD) (Santa Clara, CA, USA) was used to measure phenolic acids. Samples were run at 280 nm and 320 nm for detection of benzoic and cinnamic acid derivatives, respectively. An Agilent Zorbax ODS (4.6 × 250 mm × 5 micron) C18 column with guard column was used for separation of phenolic acids. Phenolic acids were quantified by comparison of retention times of pure analytical standards. The benzoic acid derivatives *p*-hydroxybenzoic acid, vanillic acid, and syringic acid were dissolved in methanol at the following concentrations: 100, 50, 10, 2 and 1 µg/mL, and detected on the VWD at 280 nm. The cinnamic acid derivatives caffeic acid, *p*-coumaric acid, ferulic acid, and sinapic acid were dissolved in methanol at the following concentrations 100, 50, 10, 2 and 1 µg/mL and detected on the VWD at 320 nm.

### 3.4. Complex Preparation

Phenolic acids or wheat bran extract (3 mM) and *β*-CD (3 mM) were dissolved in 50 mL H_2_O in a 1:1 ratio. The mixtures were stirred in the dark for five hours at room temperature, and then left for 12 h in the dark at room temperature. Finally, the mixture was filtered, the solution was frozen at −20 °C, and left for 24 h in a freeze drier [29,30].

### 3.5. Sample Preparation for NMR

Phenolic acids (5 mM) and *β*-CD (5 mM) were dissolved in 2 mL D_2_O and mixed by vortexing [30].

### 3.6. Sample Preparation for Mass Spectroscopy (MS)

All complexes prepared by the freeze-drying method (each 1 mg) were dissolved in a mixture of 0.8 mL water and 0.8 mL methanol [29,30].

### 3.7. Nuclear Magnetic Resonance Spectroscopy (NMR)

The ^1^H-NMR spectra were recorded by 400 MHz and 500 MHz Bruker-DRX-NMR spectrometers (Billerica, MA, USA). The NMR spectrometer was operated, and the measured data were processed by the software Top Spin. The measurement conditions were as follows: radiation with 90° pulses of 11.8 µs, 256 as a number of repetitions, and a repetition time of 13.5 s [29,30].

### 3.8. Mass Spectroscopy (MS)

The Waters’ SYNAPT G2-Si instrument (Taunton, MA, USA) was used for recording all complexes and was equipped with electron spray ionization (ESI) source and quadrupole-time of flight (Q-ToF) analyzer. The following conditions were used for recording: negative ions, resolution V-mode, rate 5 µL/min, capillary voltage 1.4 kV, cone voltage 100 V, cone gas 31 L/h, desolvation temperature 250 °C, desolvation gas (N_2_) 402 mL/h, scan time 1.0 s and inter-scan time 0.015 s. All measured data were evaluated by MassLynx software v4.1 [29,30].

### 3.9. Fourier Transform InfraRed (FT-IR) Spectroscopy

The Nicolet iS10 FT-IR spectrophotometer (Nicolet, Glendale, WI, USA) was used for recording the pure compounds-*β*-CD and phenolic acids, the physical mixture, and the complex. The following conditions were used: 64 scans, 4 cm^−1^ resolution between 4000 and 500 cm^−1^. The FT-IR spectrophotometer was operated by the software OMNIC [29,30].

### 3.10. Differential Scanning Calorimetry (DSC)

The DSC 6000 differential scanning calorimeter (by Perkin Elmer, Waltham, MA, USA) was used for studying the thermal behavior of both pure compounds, the physical mixture and the complex. Samples were dried for 24 h at 110 °C and then, the samples were weighed between 3 mg and 5 mg in aluminum pans. The following conditions were used: heating between 50 °C and 230 °C, 5 °C/min scanning rate, and 20 mL/min nitrogen flow [29,30].

### 3.11. Computational Study

A combined computational study was carried out to elaborate on the complexation mechanism of investigated structures further and *β*-CD according to the experimental results. In this study, we applied a molecular modeling study, followed by a quantitative structure–property relationship (QSPR) analysis. The initial phenolic acids’ structures were built using Avogadro software (version 1.20, http://avogadro.cc), followed by structure optimization and semiempirical quantum-mechanical calculation using MOPAC software package (MOPAC2012, http://OpenMOPAC.net). Some quantum-chemical data were taken from our previous study, where we conducted a similar study, with fewer compounds [29,30]. The current study is a logical extension of the previous study with a number of additional experiments and extended data set.

We used the parameterized model 6 (PM6) method since it has been shown to be a powerful tool in the conformational study of cyclodextrin complexes and has high computational efficiency in calculating cyclodextrin systems. Thus, PM6 uses a novel parameterization of the previously used PM3 Hamiltonian and delivers results that are comparable to the density functional theory (DFT) level [31]. Various semiempirical methods were utilized by our group in previous studies for similar systems [26,27,31]. The details on the quantitative structure–property relationship (QSPR) modeling technique used here are explained in our previous works [29,30]. The following molecular properties were calculated for each structure: the heat of formation (*H_f_*), dipole moment (*μ*), total energy (*E_t_*), and HOMO-LUMO energies. The Δ*H_f_* values for mixture systems were calculated based on Δ*H_f_* values of pure phenolic acids, using the additive formula that takes a mean value of two or three compounds, i.e., Δ*H_f_* (mix) = (Δ*H_f_* (phenolic acid 1) + Δ*H_f_* (phenolic acid 2))/2.

## 4. Conclusions

The NMR analysis illustrated the complexation between all three phenolic acids and *β*-CD based on the chemical shift differences of both inner protons H^3^ and H^5^. Additionally, this study presented that CO has the highest potential to include in the cavity of *β*-sCD in comparison to the phenolic acids FA and CA in a synthetic mixture. The MS study indicated that all three complexes between the phenolic acids and the *β*-CD are formed in different amounts. CO was included in a higher amount than CA and FA. The DSC study showed the changes in the physicochemical property of the included phenolic acid in the *β*-CD cavity. The included phenolic acid has the same melting point as *β*-CD. The FT-IR investigation showed that the complexation affects the IR activity of the phenolic acids. The bands of the included phenolic acid are shifted, or their intensity influenced, whereas the spectrum of the *β*-CD remains largely unchanged. On the whole, the spectroscopic data indicate that free phenolic acids from wheat bran could be complexed with *β*-CD. The computational study demonstrated the relationship between the binding energy and H^5^ chemical shifts of pure phenolic acids and mixture systems. The experimental and computational investigations demonstrate that the phenolic acids -CA, CO, and FA in synthetic mixtures and wheat bran extract are forming inclusion complexes with *β*-CD by non-covalent bonds. The developed QSPR model can be beneficial in assessing the type of phenolic acid mixture in the solution, as well as the concentration of presented phenolic acid compounds in the solution, with the help of additional concentration experiments. Additional studies of the pH during complex formation and isothermal titration calorimetry would also be beneficial for future work on this topic. One potential application of these complexes may be to mask undesirable flavors and improve their stability. However, this will need further study to determine the interaction of the *β*-CD in the end-product system and the efficacy through sensory studies.

## Figures and Tables

**Table 1:** ^1^H-NMR Chemical shifts (δ, ppm) for CH protons of *β*-cyclodextrin pure and their complexation with the wheat bran extract induced shifts (CIS = δ_complex_ − δ_guest_) in D_2_O at 25 °C (A) * and experimental chemical shift values (δ, H^5^) and calculated values of the enthalpy of formation (*H_f_*), Δ*H*, total energy (*E_T_*), and frontier orbital energies (*E_HOMO_*, *E_LUMO_*) for *β*-cyclodextrin complexes with phenolic acids (B) **^,†^.

**(A)**	**Extract +**	***β*-cyclodextrin**						
	**δ_free_**	**δ_complex_**	**∆δ**					
H^1^	5.0713	5.0741	−0.0028					
H^2^	3.6506	3.6517	−0.0011					
H^3^	3.9664	3.9445	0.0219					
H^4^	3.5858	3.5965	−0.0107					
H^5^	3.8621	3.8311	0.0309					
H^6^	3.8786	3.8768	0.0018					
**(B)**	**Calculated Properties of the Complex of Phenolic Acid/*β-*Cyclodextrin** **δ** **H** **^5^** **, ππ** **μ**							
**Complex**	***E_T_***		***H_f_***	**Δ** ***H*** ***_ϕ_*** **	***E_HOMO_***	***E_LUMO_***	**Δ*HL***	
CA-*β*-CD	−16,350.12		−1476.23	−9.81	−9.362	−1.203	8.159	3.764
CO-*β*-CD	−16,058.7		−1437.22	−11.72	−9.33	−1.142	8.188	3.72
FA-*β*-CD	−16,499.48		−1469.97	−9.28	−8.972	−0.893	8.079	3.791
(CA + FA)-*β*-CD	-		-	−9.55	-	-	-	3.759
(CO + FA)-*β*-CD	-		-	−10.5	-	-	-	3.731
(CA + CO)-*β*-CD	-		-	−10.77	-	-	-	3.713
(CA + CO + FA)-*β*-CD	-		-	−10.27	-	-	-	3.723

* General parameters have been used to assess each data point, and the results are reported in the SI. ** −H_f_, Δ*H_f_* is in kcal/mol, *E_T_, E_HOMO_, E_LUMO_*, and Δ*HL* are in eV. ^†^ *−*Δ*H_f_* for mixtures are calculated using an additive formula, based on Δ*H_f_* of pure phenolic acid.
